# Coarse-to-Fine Image Matching-Based Footprint Camera Calibration of the GF-7 Satellite

**DOI:** 10.3390/s21072297

**Published:** 2021-03-25

**Authors:** Lirong Liu, Junfeng Xie, Xinming Tang, Chaofeng Ren, Jiyi Chen, Ren Liu

**Affiliations:** 1 Land Satellite Remote Sensing Application Center, MNR, Beijing 100048, China; liulirong1125@163.com (L.L.); txm@lasac.cn (X.T.); whuchen@foxmail.com (J.C.); 2 College of Geological Engineering and Geomatics, Chang’an University, Xi’an 710054, China; rencf@chd.edu.cn; 3 The School of Earth Sciences and Engineering, Hohai University, Nanjing 211100, China; rs_liur@163.com

**Keywords:** GF-7 satellite, camera calibration, image matching, on-orbit

## Abstract

The GF-7 satellite is China’s first high-resolution stereo mapping satellite that reaches sub-meter resolution, equipped with new-type payloads, such as an area array footprint camera that can achieve synchronization acquisition of laser spots. When the satellite is in space, the variation of camera parameters may occur due to launch vibration and environmental changes, and on-orbit geometric calibration thereby must be made. Coupled with the data from the GF-7 satellite, this paper constructs a geometric imaging model of the area array footprint camera based on the two-dimensional direction angle, and proposes a coarse-to-fine “LPM-SIFT + Phase correlation” matching strategy for the automatic extraction of calibration control points. The single-image calibration experiment shows that the on-orbit geometric calibration model of the footprint camera constructed in this paper is correct and effective. The matching method proposed is used to register the footprint images with the DOM (Digital Orthophoto Map) reference data to obtain dense control points. Compared with the calibration result using a small number of manually collected control points, the root mean square error (RMSE) of the residual of the control points is improved from half a pixel to 1/3, and the RMSE of the same orbit checkpoints in the image space is improved from 1 pixel to 0.7. It can be concluded that using the coarse-to-fine image matching method proposed in this paper to extract control points can significantly improve the on-orbit calibration accuracy of the footprint camera on the GF-7 satellite.

## 1. Introduction

The GF-7 satellite, successfully launched on 3 November 2019, has been equipped with such payloads as bi-linear array stereo cameras, two-beam laser altimeters, as well as two area-array footprint cameras that capture laser spots synchronously. Footprint images are acquired by the CMOS (Complementary Metal Oxide Semiconductor) camera, which is characterized by relatively large noise and distortion. When the satellite is launched into space, the variation of camera parameters may occur due to the launch vibration, the inconsistence of on-orbit operating temperature and ground environment. In order to give full play to the footprint image as a “bridge” to connect the laser altimeter data and the linear array image, the image geometric quality has to be ensured and high-precision on-orbit geometric calibration has to be conducted.

Camera calibration serves to establish the corresponding relationship between the image coordinates and the object space coordinates by using the geometric imaging model, and restores each detector beam to norm. There are two main types of models: the rigorous geometric imaging model and the generalized model. The former is a conformation model to establish the remote sensing image based on the orbit, attitude of the satellite, imaging geometry and other aspects, and the rigorous models of sensors of various types are different. The latter describes the geometric relationship between the object point and the image point by directly resorting to mathematical functions such as the widely used RPC (Rational Polynomial Coefficients) model, and does not need to consider the physical meaning of sensor imaging. Moreover, the latter is characterized by relatively higher versatility and ease of use as auxiliary data of remote sensing products for users. The study and characteristic analysis of a specific sensor and the establishment of a rigorous geometric model is indispensable for the on-orbit high-precision calibration of a satellite. Due to the long focal length and narrow field of view of the GF-7 space-borne area-array camera, it is inappropriate to directly apply the calibration method of traditional aerial area-array camera, and it is necessary to explore a more effective geometric calibration model to calibrate the internal and external orientation elements of the camera and the lens distortion. Currently, the directional angle model is proven effective, which can eliminate the excessive parameterization problems of the rigorous geometric models of optical satellite cameras.

At present, the calibration research for optical satellites has mainly focused on linear array cameras. Tang et al. [1] has established a rigorous geometry model based on the space-borne optical push-broom imaging, which accurately expresses the imaging geometry of the ZY-3 satellite three-linear array camera. Many studies, such as references [2,3], also used the calibration field control data to perform on-orbit geometric calibration of the ZY-3 satellite optical linear array camera. The results show that the camera has linearity errors caused by the change of the focal length and the rotation of the CCD arrangement, and the internal distortion of the resulting image correction product can be controlled within the sub-pixels. In light of the design of the camera’s biased field of view and the high correlation of internal and external parameters, references [4,5] proposed a step-by-step solution of the external and internal orientation elements based on the directional angle model. The calculation method has been applied to the on-orbit geometric calibration experiments on ZY-1-02C and ZY-3 satellites, and achieves good results. Jiang et al. [6] presented the on-orbit geometric calibration Process of Zhuhai-1 Hyperspectral Satellites based on collinearity equation and directional angle model, and the calibration accuracy was better than 0.5 pixels.

However, there has been little research on space-borne area-array cameras. The GF-4 satellite is equipped with two area-array sensors operating in the visible electromagnetic spectrum combined with the near infrared (450–900 nm) and intermediate infrared (3.5–4.1 μm) part of the electromagnetic spectrum, with a spatial resolution of 50 and 400 m, respectively, within which many Chinese scholars have carried out related research. Wang et al. [7] proposed a rigorous geometric imaging model for an optical area array camera of GF-4 geostationary orbit satellite. The on-orbit geometric calibration was completed through two steps of external and internal calibration, the two-dimensional direction angle model was used to describe and compensate the internal distortion of the camera, and the internal accuracy of the calibrated camera was within 1 pixel. Chang et al. [8] also proposed a model based on the directional angle; the parameter calculation and the verification method of the model were provided, and the applicability of RPC model in the stationary orbit area array camera was analyzed and verified. In addition, Xu et al. [9] analyzed the key factors affecting the radiation quality and geometric accuracy of GF-4 satellite images, and introduced the construction technology of high-orbit area array imaging processing model. Experiments showed that the internal distortions of GF-4 satellite images after on-orbit geometric calibration were within 0.8 pixels along and perpendicular to the orbit. The GF-4 satellite is both a geostationary orbit satellite and a high-orbit satellite, which is about 36,000 km above the earth’s surface, while the average orbital height of the GF-7 satellite is 505 km. Both area array cameras mounted on GF-4 and GF-7 satellites have the characteristics of long focal length and narrow field of view; therefore, the method of GF-4 on-orbit calibration is of certain referential value to this study. Given the new-type area-array footprint camera of GF-7 satellite, this paper constructs an adaptive two-dimensional direction angle model for high-precision on-orbit calibration according to its data acquisition characteristics.

The calibration of external orientation elements of the image may only rely on a small number of control points, while the calibration of internal orientation elements requires a large amount of control point information as a constraint condition. However, the traditional method of setting up ground calibration fields is of high cost and poor timeliness, so the dense matching methods between satellite images and reference images, such as high-precision DOM, have become important to extract satellite geometric calibration control points. Since satellite images and reference images are often acquired by different sensors, which are also different in acquisition time, resolution, lighting condition, and viewing angle, the automatic matching technique of these multimodal remote sensing images is yet to be solved.

Commonly used remote sensing image matching methods can be divided into two categories: one is feature-based, and the other is region-based [10]. In the field of computer vision, classic feature matching algorithms such as SIFT (Scale-Invariant Feature Transform) [11], SURF (Speeded Up Robust Features) [12], FAST (Features from Accelerated Segment Test), and ORB (Oriented fast and Rotated BRIEF) [13,14] are widely used in the field of remote sensing image registration, while these descriptors are very sensitive to the radiation difference between images. In order to overcome the influence of radiation differences between multimodal images, many scholars have made improvements on the SIFT algorithm [15,16,17]. The researchers in references [18,19] replaced the gray difference gradient of the original descriptor with the ratio gradient to match the optical and synthetic aperture radar remote sensing images, which has reduced the effect of speckle noise to a certain extent.

The region-based matching method, also called template matching or correlation-based matching method, can be further divided into intensity-based correlation, frequency-domain phase correlation, and mutual information method. In recent years, more research on the regional feature descriptors constructed based on image frequency-domain phase correlation has been done, with as a theoretical basis Fourier’s theorem, which follows the basic idea of transforming the image from the spatial to the frequency domain through a Fourier transform, and then finding the relative offset between the two images by the phase correlation algorithm. The phase information contains the image translation, rotation, affine, and other transformations, which have an inhibitory effect on high-frequency noise and can better resist geometric and radiation distortion. Extensive research has been conducted on frequency-domain phase correlation matching. Leprince et al. [20] elaborated a sub-pixel level registration algorithm based on phase correlation. Wong et al. [21] introduced phase consistency into the registration of multi-source remote sensing images and achieved good results. Ling et al. [22] proposed a matching method based on phase consistency and Zernike moments; Li et al. [23] improved matching efficiency by constructing feature descriptors based on phase consistency; Ye et al. [16] constructed a directional histogram descriptor based on phase consistency and marginal information. Fan et al. [24] constructed a structure descriptor based on multi-scale weighted phase consistency. Although algorithms based on image frequency correlated with the domain phase still need further improvement to fit the scale and rotation differences between images, the domain-based matching methods, as a whole, can obtain more reliable corresponding image points between multi-source remote sensing images compared with feature-based matching methods [25].

The registration method based on phase correlation is widely used in the matching of multimodal remote sensing images because of its anti-noise and robustness. However, the phase correlation method often requires a certain projection relationship between the images to be registered based on geographic reference, that is, a preliminary geometric model of the footprint image needs to be constructed using a certain number of control points before matching. Therefore, this paper proposes a coarse-to-fine automatic matching strategy. First, the feature matching algorithm is used to obtain initial coarse matching points, and a preliminary geometric model is thereby constructed. Then, the phase matching algorithm is applied for fine matching to obtain dense and high-precision control points for the calibration of rigorous geometric imaging models.

This paper presents an on-orbit geometric calibration method for the area-array camera of GF-7. The contributions of this paper are as follows:

(1) This paper constructs a finely adjusted geometric imaging model based on the two-dimensional direction angle, which is the first on-orbit calibration study of GF-7 footprint camera to our knowledge.
(2) A coarse-to-fine “LPM-SIFT + Phase correlation” matching method is proposed for the automatic extraction of control points for calibration, which takes advantages of both feature based and phase matching. The dense controls are provided for geometric calibration without manual collection.

## 2. Materials and Methods

**Figure 1:**
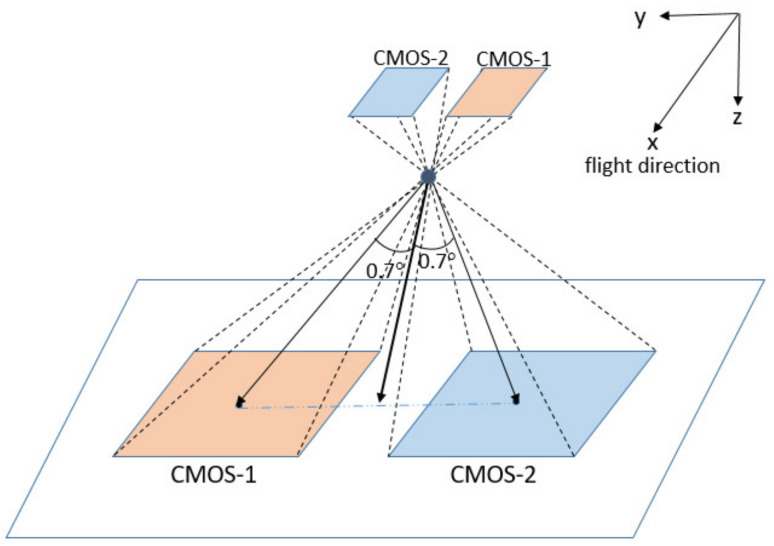
Schematic diagram of the relationship between the focal plane of the footprint camera and the ground projection.

The GF-7 satellite carries two small area-array footprint cameras using CMOS to capture ground spots of the lasers. As shown in the camera installation diagram in Figure 1, the +*X* axis is the flight direction, and the +*Z* is the optical direction axis of the receiving system. The principal optic axis of the imaging center is (0, ±0.7). The imaging FOV (Field of View) along the *x*-axis ranges from –0.1° to +0.1°, and that along the *y*-axis varies from ±0.6° to ±0.8°, which is typical of an imaging mode featuring long focal length and narrow field of view. The imaging spectrum range of the footprint camera is 500 nm to 720 nm with the pixel size of 16.5 μm × 16.5 μm and a corresponding ground resolution of 3.2 m, and the default output image size is 550 × 550 pixels with a corresponding actual ground range of 1760 m × 1760 m. As for the two area array CMOS footprint cameras, the theoretical viewing angle and detection polarity are shown in Figure 1.

**Figure 2:**
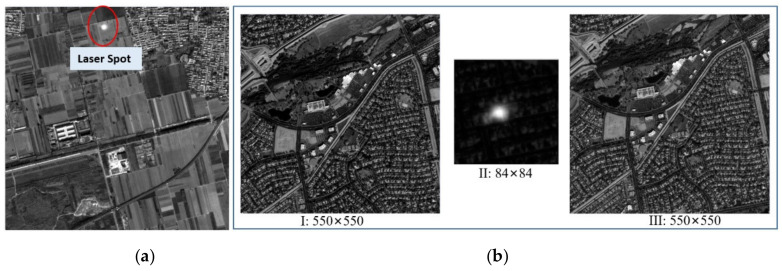
Working mode of the footprint camera. (**a**) Single exposure mode. (**b**) Mode of three exposures.

On-orbit footprint cameras can work in two modes: single exposure and three exposures. The former means that in a single imaging period (330 ms), the camera exposes once and captures a corresponding laser spot simultaneously; the latter exposes three times in a period, the image size of the first and third captures is 550 × 550 pixels with ground imagery, and the second exposure corresponds to the laser emission time, which is used to read the laser spots, i.e., images with pixels of 84 × 84. As shown in Figure 2, the overlap of the first and third footprint images obtained in the mode of three exposures reaches up to 90%, and the images obtained between the two imaging periods do not overlap in both modes.

### 2.1. Calibration Model of Two-Dimensional Direction Angles

The space-borne optical camera calibration aims to establish the correspondence between image coordinates and ground coordinates using the geometric imaging model. Therefore, the geometric imaging model is critical, and will directly affect the geometric accuracy of the image. The rigorous imaging model of an optical camera is generally a conversion formula between the image coordinates and the ground coordinates depending on the motion vector, attitude, and internal and external parameters of the satellite. Therefore, a rigorous geometric imaging model can be generally constructed as:

(1) $$\left[\begin{array}{c} X_{g}\\ Y_{g}\\ Z_{g} \end{array}\right]=\left[\begin{array}{c} X_{s}\\ Y_{s}\\ Z_{s} \end{array}\right]+\lambda *R_{J2000}^{wgs84}*R_{body}^{J2000}*\left[R_{cam}^{body}\left[\begin{array}{c} x-x_{0}-\Delta x\\ x-y_{0}-\Delta y\\ -f \end{array}\right]+\left[\begin{array}{c} d_{x}\\ d_{y}\\ d_{z} \end{array}\right]\right]$$

where ${\left[\begin{array}{ccc} X_{g} & Y_{g} & Z_{g} \end{array}\right]}^{T}$ represents the object space coordinate in the *WGS84* coordinate system, ${\left[\begin{array}{ccc} X_{s} & Y_{s} & Z_{s} \end{array}\right]}^{T}$ represents the satellite body’s coordinate in the *WGS84* coordinate system, $R_{cam}^{body}$ represents the installation matrix from the camera coordinate system to the satellite body coordinate system, *J*2000 is an Earth-centered inertial (ECI) coordinate reference frame, $R_{J2000}^{body}$ represents the rotation matrix from the *J*2000 to the body coordinate system, $R_{wgs84}^{J2000}$ denotes rotation matrix from the *WGS84* to the *J*2000 coordinate system, ${\left[\begin{array}{ccc} d_{x} & d_{y} & d_{z} \end{array}\right]}^{T}$ is the origin deviation between the camera and body coordinate system, $\left(x_{0},y_{0}\right)$ is the principal point, f is the focal length, $\left(\Delta x,\Delta y\right)$ is the internal systematic error parameters, and λ is proportional coefficient.

The geometric model of the GF-7 satellite area array footprint camera is different from both the traditional linear array CCD of the push-broom satellite and that of the aerial area array camera. We need to analyze the factors that affect the geometric quality of the images coupled with the imaging conditions and operating environment. The on-orbit calibration of the footprint camera is mainly carried out in terms of the angle error of installation, the internal system error of the camera and the optical distortion, etc. Under ideal circumstances, when the camera is installed on a satellite, the three-axis directions of the camera and the satellite body coordinate system should be in strict consistency. However, in the actual installation process, there is an angular difference between the three axes of the two coordinate systems, called the angular error in camera installation. Before the satellite gets launched, although the camera installation angle is calibrated in ground laboratory, the angle will change due to various factors, such as stress release, material outgassing, and space environment changes during the launch [9]. Considering that the satellite camera is far from the ground, even very small angle errors have a great influence on the positioning accuracy. It is necessary to calculate the camera installation angle error through on-orbit calibration to accurately determine the rotation matrix between the camera coordinate system and the satellite system in the geometric imaging model. In addition, due to the complex space environment, the cameras on satellite must be re-calibrated regularly.

**Figure 3:**
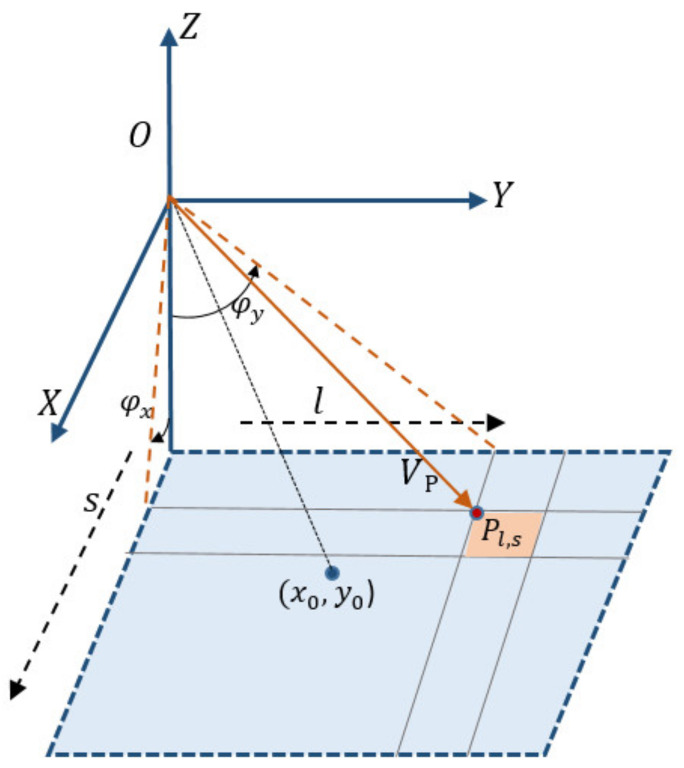
Two-dimensional detector directional angle of the area-array camera.

The internal orientation elements describe the conversion from the image plane to the camera coordinate system, and determine the light ray vector of each detector. This paper constructs a two-dimensional detector directional angle model as the internal calibration model for the GF-7 footprint camera. As shown in Figure 3, OXYZ denotes the space auxiliary camera coordinate system, O denotes the camera projection center, $\left(x_{0},y_{0}\right)$ denotes the principal point, $\left(s,l\right)$ is the detector’s image plane coordinate, $V_{P}$ is the viewing direction of point $P_{l,s}$, and $\varphi_{x}$ and $\varphi_{y}$ are the directional angles.

The tangents of directional angles are defined as follows:

(2) $$\left\{\begin{array}{c} \tan\varphi_{x}=\frac{x-x_{0}-\Delta x}{f}\\ \tan\varphi_{y}=\frac{y-y_{0}-\Delta y}{f} \end{array}\right.$$

Then, the LOS (Line of Sight) of each detector in the camera coordinate system can be determined by solving the directional angles. The geometric calibration model of the footprint camera based on the two-dimensional direction angle constructed in this paper is shown as follows:

(3) $$\left[\begin{array}{c} \tan\varphi_{x}\\ \tan\varphi_{y}\\ -1 \end{array}\right]=\lambda *R_{U}*R_{body}^{cam}*\left[R_{J2000}^{body}*\left[R_{wgs84}^{J2000}*\left[\begin{array}{c} X_{g}-X_{s}\\ Y_{g}-Y_{s}\\ Z_{g}-Z_{s} \end{array}\right]\right]-\left[\begin{array}{c} d_{x}\\ d_{y}\\ d_{z} \end{array}\right]\right]$$

(4) $$R_{U}=\left[\begin{array}{ccc} a_{1} & a_{2} & a_{3}\\ b_{1} & b_{2} & b_{3}\\ c_{1} & c_{2} & c_{3} \end{array}\right]=\left[\begin{array}{ccc} cos\Delta \varphi & 0 & -sin\Delta \varphi \\ 0 & 1 & 0\\ sin\Delta \varphi & 0 & cos\Delta \varphi \end{array}\right]\left[\begin{array}{ccc} 1 & 0 & 0\\ 0 & cos\Delta \omega & -sin\Delta \omega \\ 0 & sin\Delta \omega & cos\Delta \omega \end{array}\right]\left[\begin{array}{ccc} cos\Delta k & -sin\Delta k & 0\\ sin\Delta k & cos\Delta k & 0\\ 0 & 0 & 1 \end{array}\right]$$

where $R_{U}$ is the setting matrix, and the installation error angles $\left(\Delta \varphi ,\Delta w,\Delta k\right)$ of the $R_{U}$ matrix can be solved to correct the deviation of exterior orientation of each image.

A stepwise calibration is performed for the external and internal parameters estimation.

According to Equation (3), we set:

(5) $$\left[\begin{array}{c} U_{x}\\ U_{y}\\ U_{z} \end{array}\right]\;=\;R_{body}^{cam}*\left[R_{J2000}^{body}*\left[R_{wgs84}^{J2000}*\left[\begin{array}{c} X_{g}-X_{s}\\ Y_{g}-Y_{s}\\ Z_{g}-Z_{s} \end{array}\right]\right]-\left[\begin{array}{c} d_{x}\\ d_{y}\\ d_{z} \end{array}\right]\right]$$

Then, Equation (3) can be transformed into error Equation (6) for external calibration:

(6) $$\left\{\begin{array}{c} F\left(X_{e},X_{i}\right)=-\frac{a_{1}U_{x}+a_{2}U_{y}+a_{3}U_{z}}{c_{1}U_{x}+c_{2}U_{y}+c_{3}U_{z}}-\tan\left(\varphi_{x}\right)\\ G\left(X_{e},X_{i}\right)=-\frac{b_{1}U_{x}+b_{2}U_{y}+b_{3}U_{z}}{c_{1}U_{x}+c_{2}U_{y}+c_{3}U_{z}}-\tan\left(\varphi_{y}\right) \end{array}\right.$$

Then, the error equation is linearized, and the external parameters in the $R_{U}$ matrix can be solved by least squares method using a certain number of ground control points.

As the directional angle of each detector is different, if Equation (3) is directly used to solve the directional angles of each detector of the camera, a large number of ground control points are needed, which is difficult to operate in practice. Polynomial model is used to model the tangent of the directional angles in our study. As the internal distortion is low order because of the narrow FOV of footprint camera, the cubic polynomial, which has high orthogonality and low correlation [7], is adopted as the internal calibration model:

(7) $$\left\{\begin{array}{c} \;tan\varphi_{x}=a_{0}+a_{1}\cdot s+a_{2}\cdot l+a_{3}\cdot s\cdot l+a_{4}\cdot s^{2}+a_{5}\cdot l^{2}+a_{6}\cdot s^{2}\cdot l+a_{7}\cdot s\cdot l^{2}+a_{8}\cdot s^{3}+a_{9}\cdot l^{3}\\ tan\varphi_{y}=b_{0}+b_{1}\cdot s+b_{2}\cdot l+b_{3}\cdot s\cdot l+b_{4}\cdot s^{2}+b_{5}\cdot l^{2}+b_{6}\cdot s^{2}\cdot l+b_{7}\cdot s\cdot l^{2}+b_{8}\cdot s^{3}+b_{9}\cdot l^{3} \end{array}\right.$$

where $\left(a_{0},a_{1},a_{2}\ldots a_{9},b_{0},b_{1},b_{2}\ldots b_{9}\right)$ are polynomial coefficients and $\left(s,l\right)$ are the detector’s image plane coordinates.

According to Equation (7), a certain number of uniformly distributed control points can be applied to calculate the corresponding directional angles, after which the 20 coefficients of the polynomial are solved by the least-square method to obtain the directional angles.

### 2.2. Extracting Control Points by Coarse-to-Fine Image Matching

The feature-based matching method can achieve automatic matching of two images without any initial conditions, while the phase correlation-based registration method requires the projection relationship established between the images based on geographic reference. In order to accomplish the automatic matching of footprint images and DOM reference images to obtain dense control points, this paper proposes a coarse-to-fine matching strategy while making full use of the advantages of feature-based and region-based matching methods. First, the SIFT based Locality Preserving Matching (LPM-SIFT) [26] method is applied to initially obtain a small number of matching points, and a preliminary geometric model is thereby constructed. Then, the phase correlation algorithm for fine matching is applied to obtain dense and high-precision control points for the calibration of rigorous geometric imaging models.

**Figure 4:**
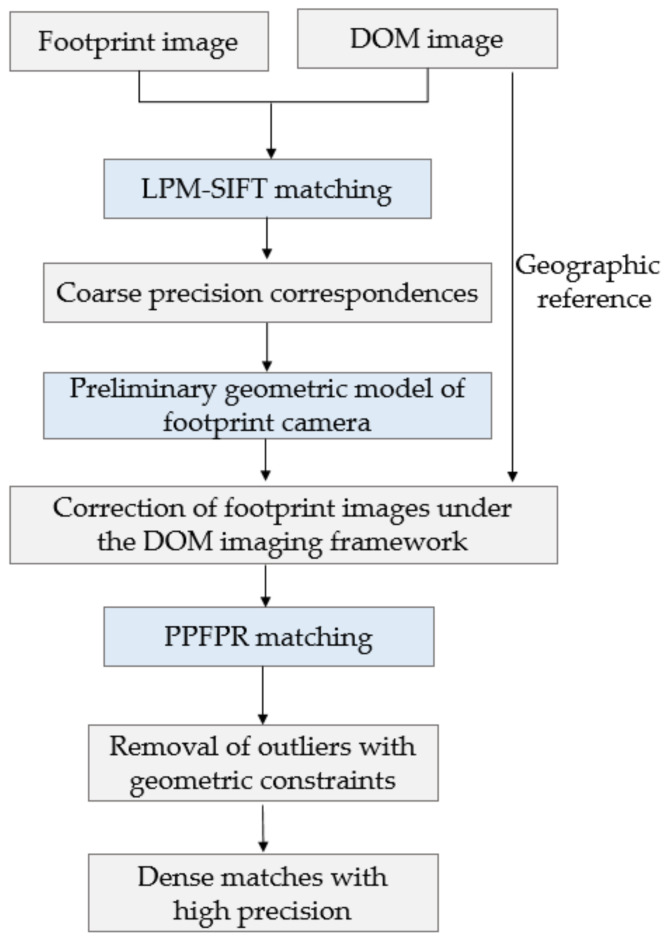
The overall matching process of the footprint image and the reference images.

The method proposed in this paper can be divided into three steps, as shown in Figure 4. First, the LPM-SIFT matching algorithm is applied to extract *m* coarse corresponding image points, and then a preliminary RPC model or rigorous geometric model of the footprint images based on the *m* points is initially constructed. Based on this model, each pixel and its neighborhood of the footprint image can be projected to the DOM reference image, and then the multi-level pyramid phase matching in frequency domain based on probability relaxation algorithm (PPFPR) is applied to further extract dense corresponding points.

#### 2.2.1. LPM-SIFT

Feature-based image matching generally consists of two main steps. First, a feature operator is used to extract a set of preliminary matching point pairs, and then, geometric constraints are applied to remove outliers. A SIFT feature operator can serve to keep rotation, scaling, and brightness invariant, and also ensure the stability of change of view, affine transformation, and noise. Therefore, the SIFT operator is adopted in this paper to extract feature points. The traditional SIFT uses a 128-bit feature descriptor, taking the optimal matching point with smallest Hamming distance, and KD-Tree is used to search the nearest neighbor feature point.

Traditional SIFT may produce a certain amount of repeated matching points, one-to-many matching points, and wrong matching points. To obtain reliable correspondences between two feature sets, the mismatch removal approach is critical. The RANSAC algorithm is classic in this respect, in order to improve the accuracy and speed of outliers removal task, this study uses the advanced LPM (Locality Preserving Matching) approach [26].

LPM is a novel mismatch removal method for feature matching. The main goal of LPM is to remove the outliers to establish accurate correspondences, the principle of which is to maintain the local neighborhood structures of the potential true matches. An improved cost function which preserves the consensus of neighborhood topology is designed to solve the problem.

If the image pair are ideal rigid transformation and the distance between any feature correspondence is constant, the cost function *C* is defined as:

(8) $$C\left(T;S,\lambda \right)=\underset{i\in T}{\sum }\underset{j\in T}{\sum }\;{\left(d\left(x_{i},x_{j}\right)-d\left(y_{i},y_{j}\right)\right)}^{2}+\lambda \left(N-\left|T\right|\right)$$

(9) $$s=\left({\left\{x_{i},x_{j}\right\}}_{i}^{N}\right)=1$$

where $s=\left({\left\{x_{i},x_{j}\right\}}_{i}^{N}\right)=1$ denotes a set of N putative feature correspondences extracted from two given images, d is the Euclidean distance metric, T denotes the unknown inlier point set, and $\left|\;\cdot \;\right|$ denotes the cardinality of a set. Ideally, the first term of C should be zero.

Considering preserving the local neighborhood structures among feature points, the cost function in Equation (8) can be adjusted as:

(10) $$C\left(I;S,\lambda \right)=\underset{i\in I}{\sum }\frac{1}{2K}\left(\underset{j|x_{j}\in N_{x_{i}}}{\sum }\;{\left(d\left(x_{i},x_{j}\right)-d\left(y_{i},y_{j}\right)\right)}^{2}+\underset{j|y_{j}\in N_{y_{i}}}{\sum }\;{\left(d\left(x_{i},x_{j}\right)-d\left(y_{i},y_{j}\right)\right)}^{2}\right)+\lambda \left(N-\left|I\right|\right)$$

where $N_{x}$ denotes the neighborhood of point x, K denotes the number of neighbors. A simple strategy that searches the K nearest neighbors for each point in the corresponding point set under the Euclidean distance is adopted. The first term 1/2K in Equation (10) is used to normalize the contribution of each element in the neighborhood.

As demonstrated in extensive experiments on various image pairs, compared with other state-of-the-art methods, LPM performs better both in accuracy and time cost [26], which can accomplish the mismatch removal from thousands of candidate correspondences in only a few milliseconds.

#### 2.2.2. PPFPR Matching

In view of the characteristics of the satellite remote sensing, a small number of coarse correspondences of the footprint image and the DOM reference image obtained by LPM-SIFT method can be preliminarily applied to calculate the preliminary geometric model parameters of the camera; the scale, direction, and positioning difference between the footprint image of the GF-7 satellite and the DOM reference image can be determined by the imaging parameters and geographic reference information, and then the phase matching method based on probability relaxation algorithm [27] is employed in our study to obtain dense matches.

**Figure 5:**
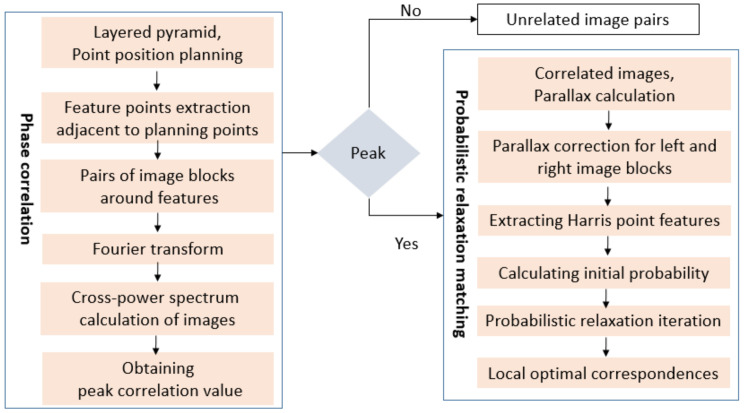
PPFPR matching workflow.

Based on the geometric model of the footprint image and the DOM image, PPFPR method constructs a relative correction model, correcting the footprint image under the DOM framework to reduce the influence of image rotation, scaling, and translation between the image pair. Then, a multi-level pyramid phase matching method is adopted, which uses a HARRIS feature operator to extract corner points to obtain a uniformly distributed pixel-level matching point set; after the calculation of the phase correlation coefficient, the parallax of the original image pairs can be calculated. Coupled with the probabilistic relaxation algorithm, the local optimal correspondences with sub-pixel accuracy are obtained, after which the least-square criterion is used to further refine the matches. The matching process is shown in Figure 5.

### 2.3. Design of the Experiments

To verify the performances of the on-orbit geometric calibration model constructed and the coarse-to-fine matching method proposed in this paper, the calibration and verification experiments based on manual and automatic matching control points were carried out, respectively. For the calibration experiments, a certain number of control points were used to solve the external and internal parameters of the footprint camera according to the two-dimensional direction angle model established in Section 2.1. A stepwise calibration process using least-square iterative was performed for parameter estimation: external parameters were first estimated, and then internal parameters were estimated.

#### 2.3.1. Verification Method

To verify the calibration accuracy, the method of single-image checkpoint verification in image space is adopted to verify the calibration result.

Primarily, an image $A_{0}$ from a certain orbit is chosen for calibration, where a certain number of control points are applied to obtain its internal and external calibration parameters according to the two-dimensional direction angle model proposed. Then, other images $A_{1},A_{2}\ldots A_{n}$ from the same orbit acquired by the same CMOS camera or images $B_{1},B_{2}\ldots B_{n}$ from different orbits are chosen to perform accuracy verification. The verification process can be elaborated as: selecting a certain number of checkpoints on the verification image and calculating the directional angles *φ_x_* and *φ_y_* corresponding to each checkpoint by internal calibration formula (7) acquired by image $A_{0}$. Then, the corresponding image coordinates of directional angles are calculated using the inverse calculation model (11) as follows:

(11) $$\left\{\begin{array}{c} \;x=a_{0}+a_{1}\cdot \varphi_{x}+a_{2}\cdot \varphi_{y}+a_{3}\cdot \varphi_{x}\cdot \varphi_{y}+a_{4}\cdot \varphi_{x}{}^{2}+a_{5}\cdot \varphi_{y}{}^{2}+a_{6}\cdot \varphi_{x}{}^{2}\cdot \varphi_{y}+\\ a_{7}\cdot \varphi_{x}\cdot \varphi_{y}{}^{2}+a_{8}\cdot \varphi_{x}{}^{3}+a_{9}\cdot \varphi_{y}{}^{3}\\ y=b_{0}+b_{1}\cdot \varphi_{x}+b_{2}\cdot \varphi_{y}+b_{3}\cdot \varphi_{x}\cdot \varphi_{y}+b_{4}\cdot \varphi_{x}{}^{2}+b_{5}\cdot \varphi_{y}{}^{2}+b_{6}\cdot \varphi_{x}{}^{2}\cdot \varphi_{y}+\\ b_{7}\cdot \varphi_{x}\cdot \varphi_{y}{}^{2}+b_{8}\cdot \varphi_{x}{}^{3}+b_{9}\cdot \varphi_{y}{}^{3} \end{array}\right.$$

where x,y are the estimated values of the image coordinates of checkpoints, assuming that $x^{0},y^{0}$ are the image coordinates of the checkpoints manually collected or obtained by target positioning algorithm on the original image. The residuals of image coordinates are calculated as follows:

(12) $$\left\{\begin{array}{c} d_{x}=x^{\prime }-x^{0}\\ d_{y}=y^{\prime }-y^{0} \end{array}\right.$$

To assess the calibration result, the universally agreed standard root mean square error (RMSE) is used as the evaluation metric. The *RMSE* of n checkpoints are obtained by Equation (13):

(13) $$RMSE=\sqrt{\frac{\sum_{i=1}^{i=n}\left(d_{xi}^{2}+d_{yi}^{2}\right)}{n}}$$

#### 2.3.2. Design of the Calibration and Verification Experiments

To validate the performance of the proposed geometric calibration model, GF-7 footprint images of orbit 154 and orbit 245 covering Beijing area in China and California area in America were collected. The data were acquired by footprint camera 1 in single exposure mode during the on-orbit test phase of the GF-7 satellite in 2019. Several images from the two orbits were chosen for single-image calibration experiment based on some manual collected control points, and the verification experiment of images from the same orbit and different orbital were carried out respectively.

**Figure 6:**
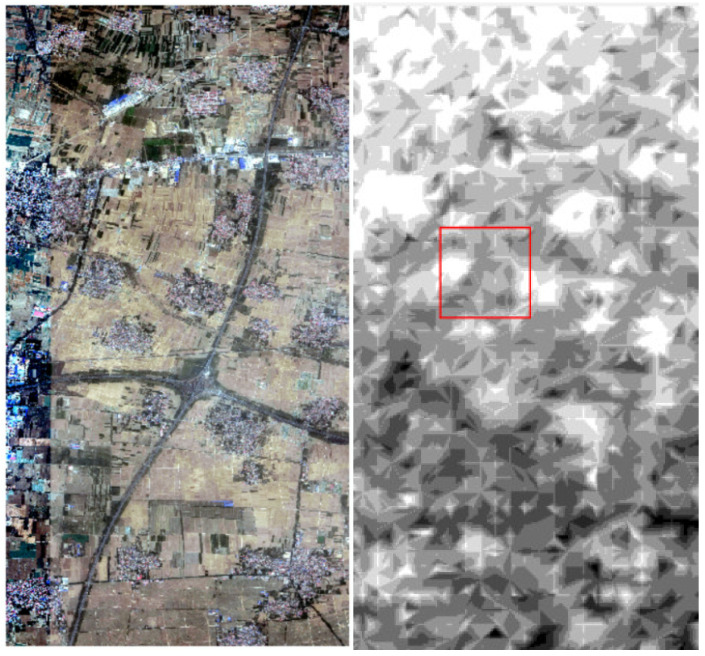
DOM (0.5-m resolution) and DEM (90-m grid) of SuperView-1.

To verify the effect of the coarse-to-fine matching method, the calibration-verification experiment based on dense matches was designed. In the experiment, the footprint images of orbit 154 covering Beijing area were chosen for the “LPM-SIFT + Phase correlation” matching process. The LPM-SIFT matching was carried out firstly, followed by PPFPR matching. As shown in Figure 6, the reference data are the DOM produced by SuperView-1 satellite images with 0.5-m resolution, and the DEM data with 90-m grid was also applied to assist in extracting rough elevation values of the matching points.

After the matching process, images from orbit 154 and the dense matching points were utilized for single-image calibration and verification experiments. Comparison and analysis are made between the calibration results based on manual control points and automatic matching points.

## 3. Results and Discussion

### 3.1. Single-Image Verification Results Based on Manual Controls

**Figure 7:**
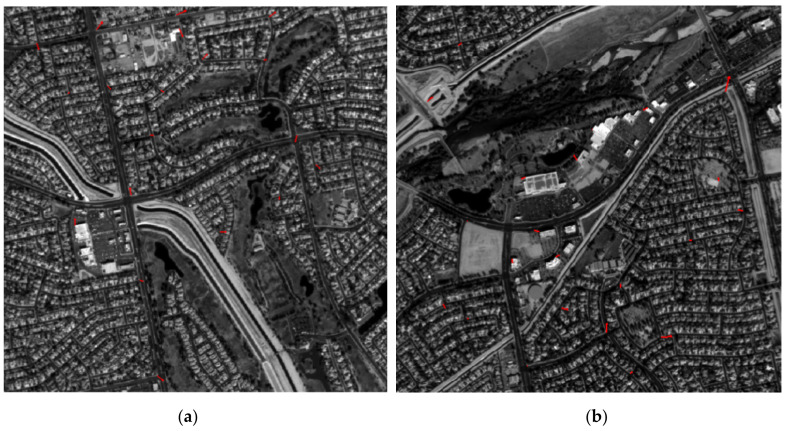

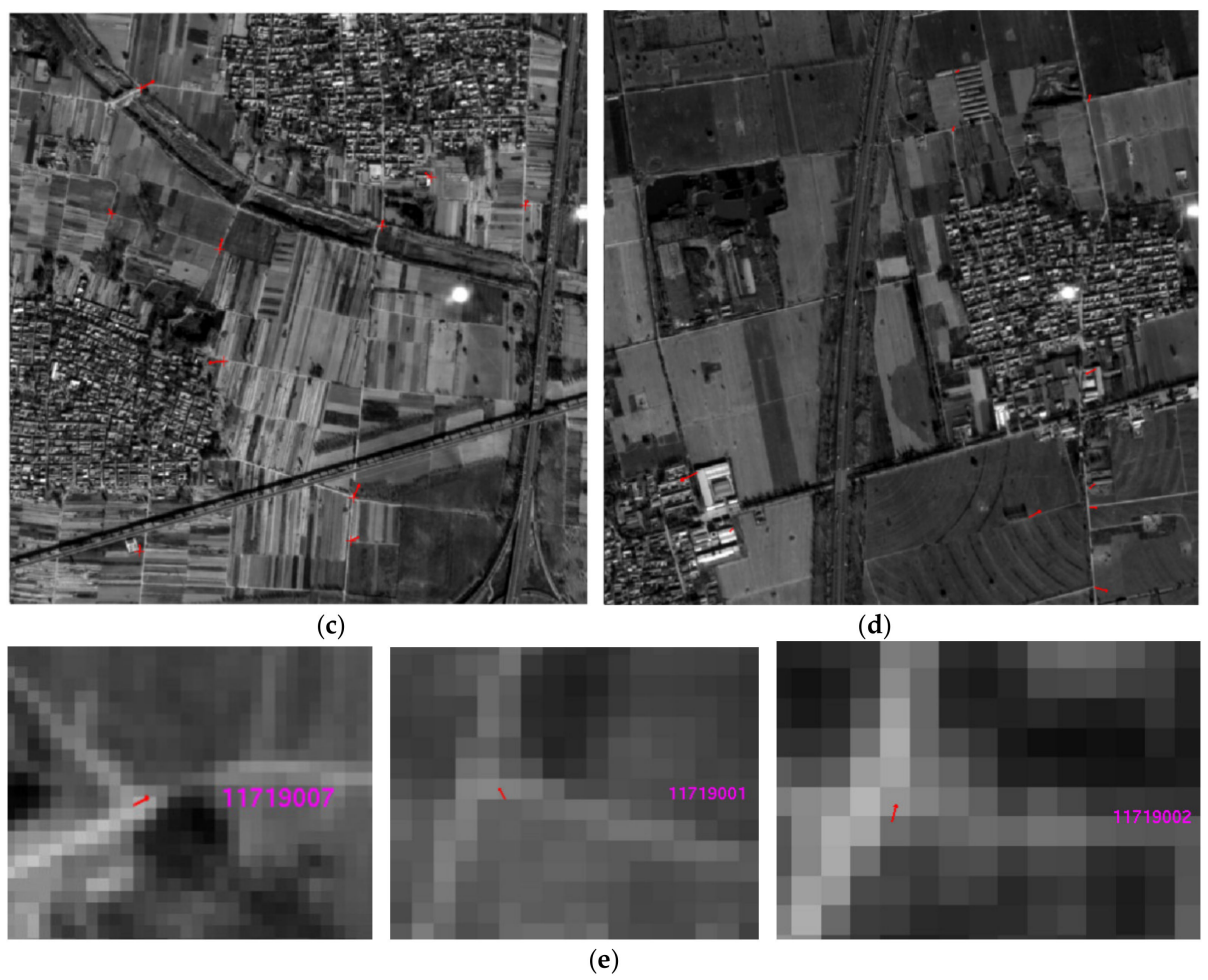
Residual distribution of checkpoints on the footprint images. (**a**) RMSE of Image 661: 0.95 pixel. (**b**) RMSE of Image 664: 0.87 pixel. (**c**) RMSE of Image 719: 0.98 pixel. (**d**) RMSE of Image 721: 0.98 pixel. (**e**) Details of some checkpoints’ residuals on image 719.

The images 719,721 from orbit 154 and images 661,664,713 from orbit 245 were chosen for single-image calibration experiment, and 25 manually collected control points for each image were used for calibration. As shown in Table 1, the RMSE of residuals of the control points is about 0.5 pixel. A small number of checkpoints on images from the same orbit were also collected for verification. In order to ensure a certain distance between the verification area and the calibration area, the ground distance between the image number 713 is more than 100 km away from the image number 669 in Orbit 245. The checkpoint residuals listed in Table 1 show that the overall accuracy of the single-image calibration and verification in the same orbit by manually collected controls is within 1 pixel. The residual distributions of the experiment images are shown in Figure 7; the point residuals are all magnified by 10 times for clarity.

**Figure 8:**
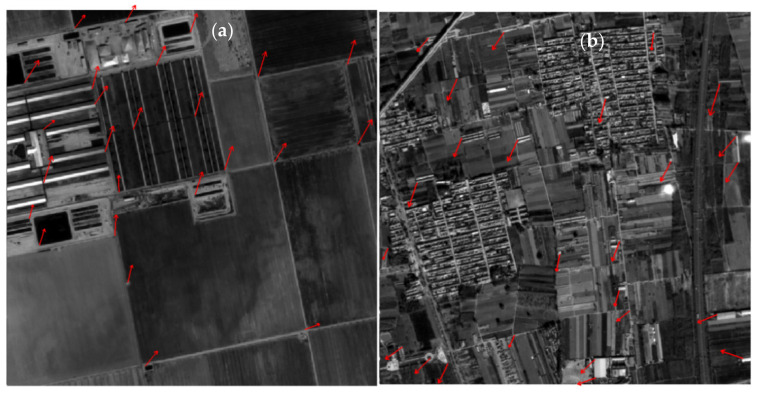
Residuals of the verification results of different orbit data (10× magnification). (**a**) RMSE of Image 669: 3.25 pixels. (**b**) RMSE of Image 717: 3.17 pixels.

Furthermore, the calibration accuracy was verified by images of different regions from other orbits. The data of orbit 154 covering Beijing area and 245 covering California were used to verify each other. As shown in Table 2, Beijing→California denotes that the data covering the Beijing area was used for calibration and the data covering California for verification, and vice versa. Results show that the RMSEs of checkpoints on images from diverse orbits are up to 3 pixels. It can be seen from the residual distribution of checkpoints in Figure 8, there are obvious systematic errors between the data of orbit 245 and 154, which may be caused by various factors, such as different time, location, atmospheric environment, and ground objects, for obtaining data, as well as the difference in the accuracy of attitude and orbit data. In addition, due to the weak stability of the GF-7 satellite in operation during the on-orbit test phase, the calibration parameters such as the installation error angles calculated by one orbit data cannot be directly applied to the error correction task for the other orbit data. The systematic errors can be eliminated by adding some ground controls as an auxiliary in the verifying images.

### 3.2. Calibration-Verification Results Based on Dense Matches

**Figure 9:**
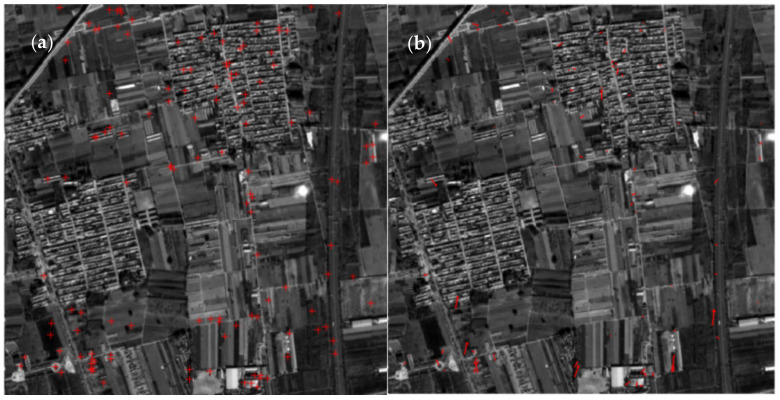

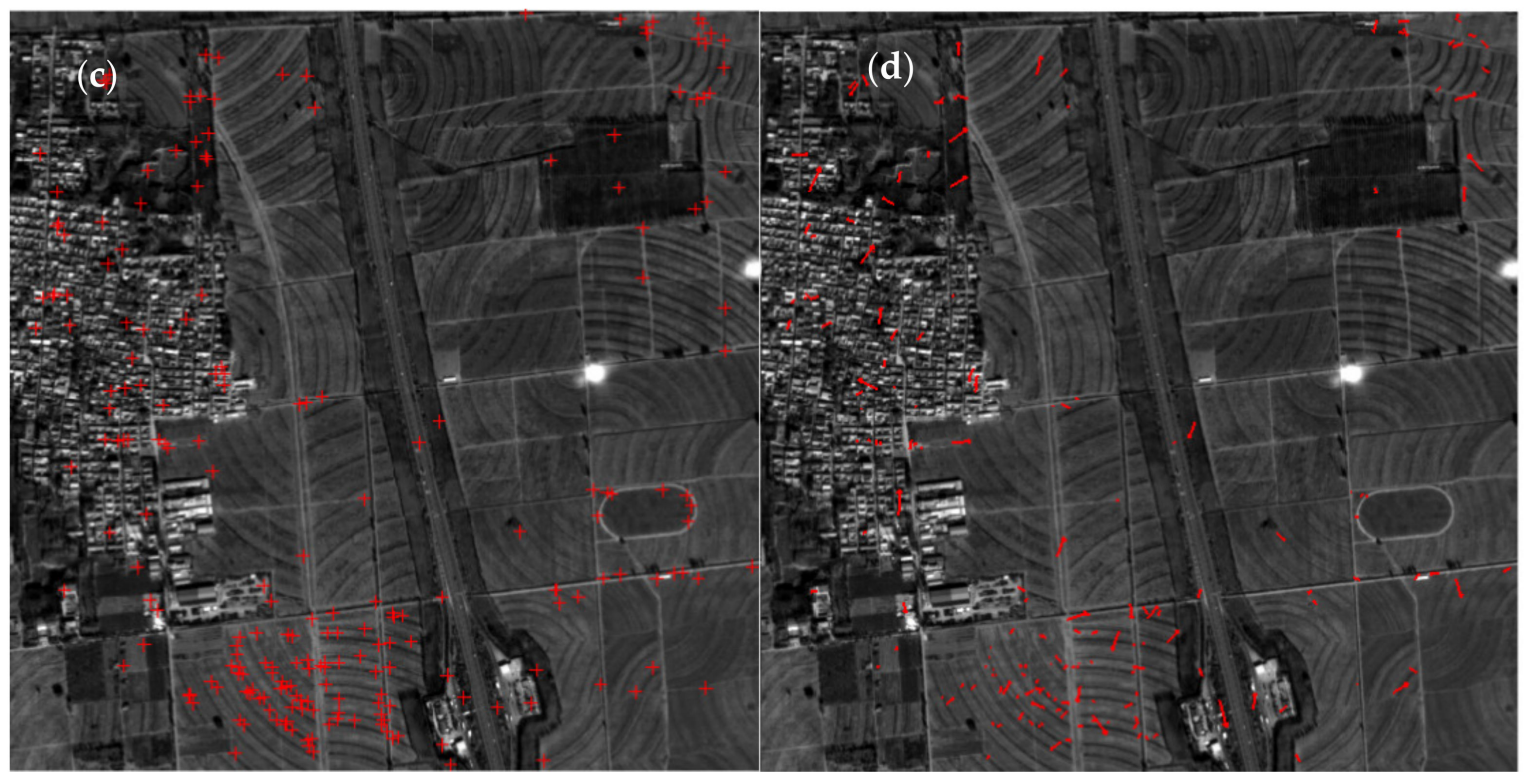
LPM-SIFT matching results. (**a**) 142 original matches on image 717. (**b**) Residual distribution of image 717 after mismatch removal (10× magnification). (**c**) 223 original matches on image 723. (**d**) Residual distribution of image 223 after mismatch removal (10× magnification).

In this experiment, the footprint images 717,723 from orbit 154 were chosen for the two-step matching. Based on LPM-SIFT method, images 717 and 723 were matched with the DOM, respectively. As the results shown in Table 3, the number of initial matches is 142 and 223, respectively. However, it is unavoidable to get some gross errors in feature-based matching. Thus, the geometric calibration model of the camera was used to verify the residuals of matches in this study. The matching points were used as control points, with a view to removing those correspondences with large errors. Figure 9 shows the initial matches (Figure 9a,c) and the residual distribution after mismatch removal (Figure 9b,d) of the test images. On the whole, the correspondences obtained by the LPM-SIFT algorithm are not dense enough and show a relatively uneven distribution. Even though many mismatches have been eliminated, the RMSE of the LPM-SIFT matching points is about 1–2 pixels. Thus, the phase correlation matching should be applied to obtain dense matching points.

**Figure 10:**
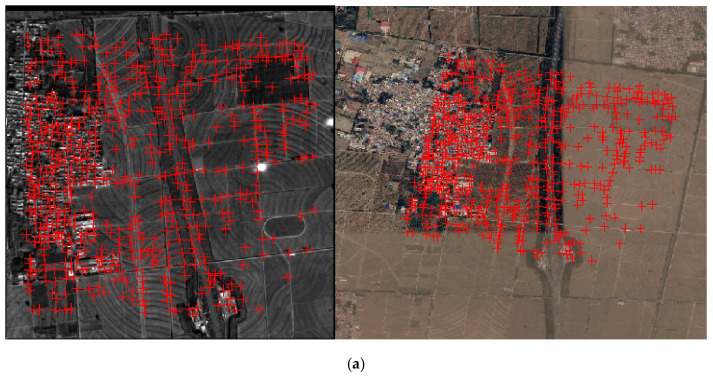

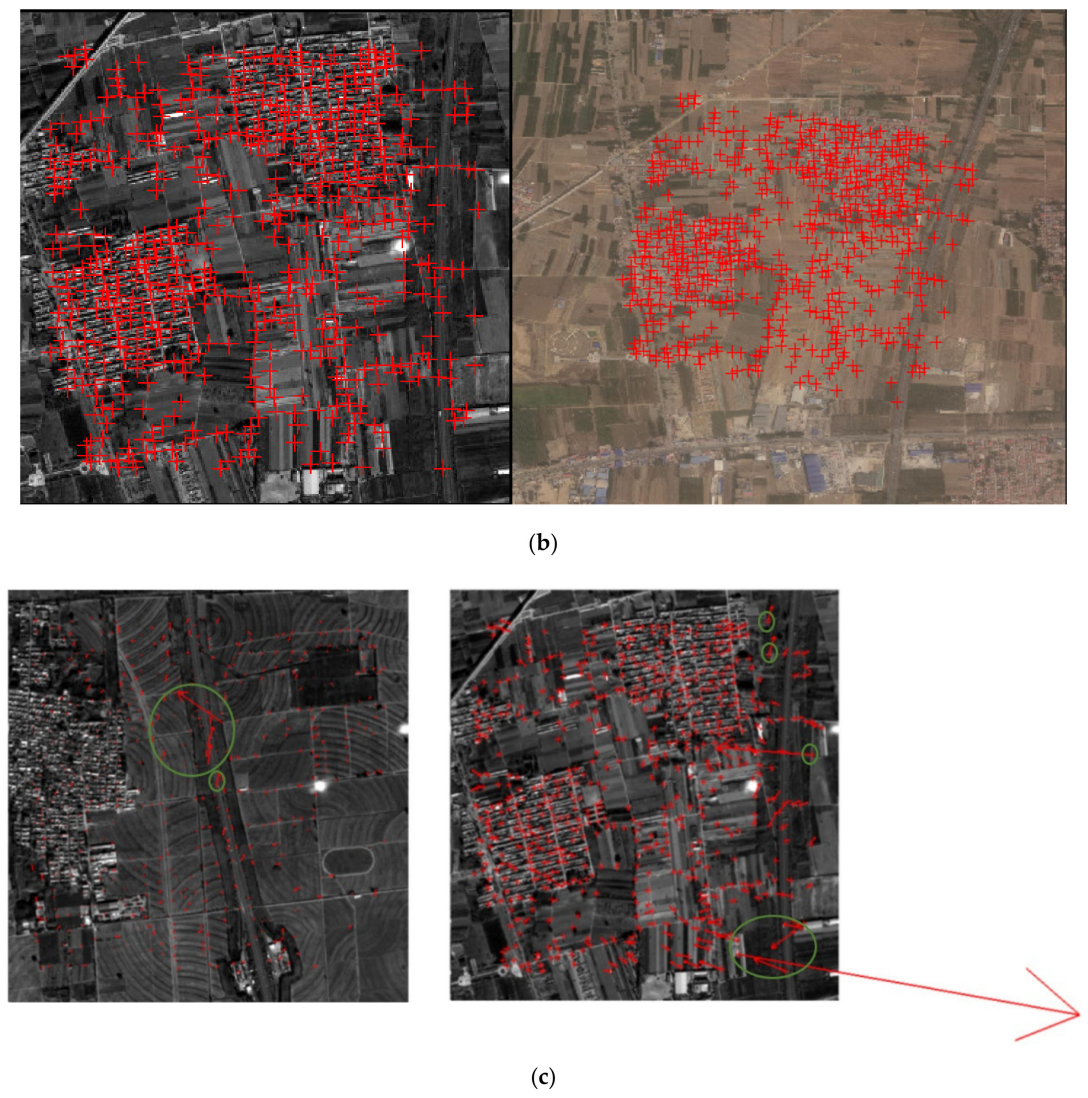
Initial matching results of the PPFPR algorithm. (**a**) 584 matches between image 723 and SuperView-1 DOM. (**b**) 586 matches between image 717 and SuperView-1 DOM. (**c**) Residual distributions of initial matches on images 723 and 717.

Based on the control points obtained by the LPM-SIFT matching, the parameters of RPC model of the footprint camera can be calculated preliminarily, and then the PPFPR method was carried out. The matching results are shown in Table 3 and Figure 10. The initial matches on images 723 and 717 were 584 and 586, respectively. As shown in Figure 10c, the residual of each point was verified by the geometric calibration model, several points with gross errors larger than three times of the RMSE have been eliminated. The final correspondences of PPFPR matching are all over 580 with a relatively even distribution.

**Figure 11:**
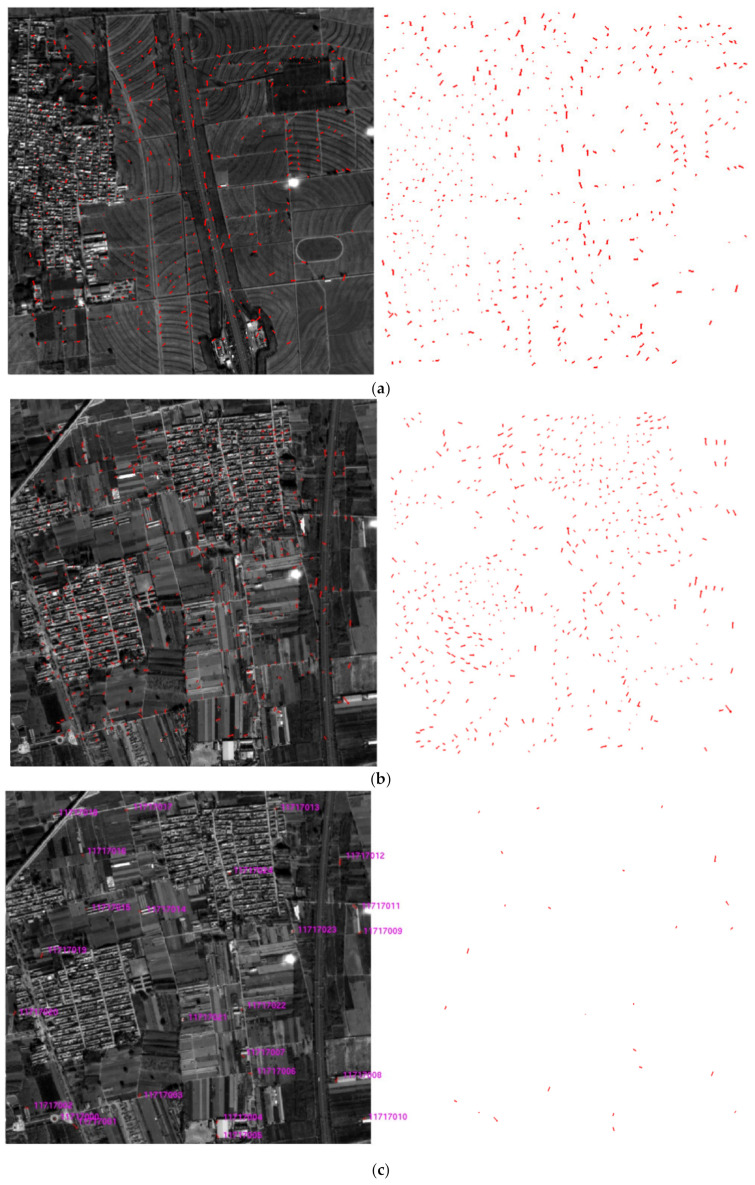
Final matches and residual distributions (10× magnification). (**a**) 580 points left after mismatch removal on image 723 (RMSE: 0.36 pixel). (**b**) 581 points left after mismatch removal on image 717 (RMSE: 0.39 pixel). (**c**) Residual distribution of 25 manually collected control points on image 717 (RMSE: 0.55 pixel).

Through the coarse-to-fine “LPM-SIFT + Phase correlation” matching process, images 717 and 723 obtained 581 and 580 control points, respectively, which were used for single–image geometry calibration. Experiment results in Table 3 show that the RMSE of the dense control points is close to 1/3 pixel in image space, while the RMSE is 0.55 pixel when using 25 manually collected control points. Figure 11a,b show the final matches and residual distributions on images 723 and 717, Figure 11c shows the residual distribution of 25 manually collected control points on image 717, the point residuals in Figure 11 were all magnified by 10 times for clarity. It can be concluded that the matching method proposed in our study is feasible and effective, which is conducive to the improvement of the overall accuracy.

**Figure 12:**
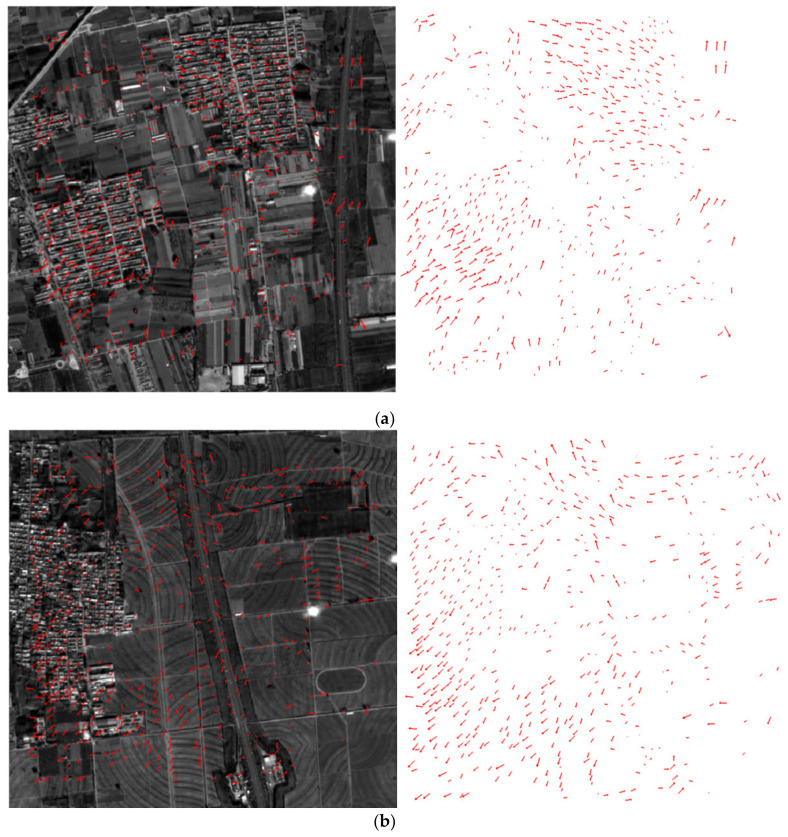
Verification results based on dense matches (10× magnification). (**a**) Residual distributions of 581 checkpoints on image 717 (RMSE: 0.67 pixel). (**b**) Residual distributions of 580 checkpoints on image 723 (RMSE: 0.68 pixel).

To further evaluate the performance of the proposed coarse-to-fine image registration method, the same orbit verification experiment in image space was conducted based on the dense matches. In the experiment, mutual verifications between images 717 and 723 from orbit 154 were carried out. The results in Table 4 show that the RMSE of the dense checkpoints are 0.67 and 0.68 pixels, respectively, and the residual distributions are shown in Figure 12.

Compared to the verification results by utilizing manually collected controls in Table 1, the accuracy of checkpoints has been improved from 1 pixel to less than 0.7 pixel, indicating that the proposed method can produce high-quality and dense control points in even distribution. The matching results can effectively improve the on-orbit calibration accuracy of the space-borne footprint camera on GF-7 satellite.

### 3.3. Discussion

The footprint camera of GF-7 satellite images the ground at the time of laser altimeter emission; the position of laser spot center can be determined via the footprint image, and the footprint image can also be matched with the high-resolution images of the same area taken by the bi-linear array camera. Therefore, the footprint image serves as a “bridge” to connect the laser spot data and the linear array image; the positioning accuracy of the footprint camera is critical to the whole payload system of the GF7 satellite. As an area array footprint camera is used, and there is no overlapping area between the adjacent images, the on-orbit calibration and verification experiments are all based on a single image in this study.

The experimental results can be seen in Section 3: based on the two-dimensional direction angle model proposed, the verification accuracy of the same orbit data using manual controls is within 1 pixel in image space, and the corresponding ground positioning accuracy is better than 3 m. Based on the “LPM-SIFT + Phase correlation” matching strategy, more than 580 matching points can be automatically obtained on each image for calibration, and the verification accuracy has been improved to less than 0.7 pixel, which is about 2 m on the ground. Compared with using the manual collected controls, the coarse-to-fine matching method is conducive to the improvement of calibration accuracy.

In the matching experiment, it is shown that the footprint and DOM images that need to be matched are multimodal and have big difference in resolution, lighting condition, viewing angle, etc. After LPM-SIFT matching, only about 100 matches with uneven distribution were obtained, and the verification accuracy is over 1 pixel. Furthermore, these matching points are mainly concentrated in areas with obvious features and textures in the image, which is determined by the principle and characteristics of the feature matching algorithm. After PPFPR matching, the correspondences are much more dense and evenly distributed. The results of the calibration and verification experiments show that these dense matching points can be effectively used for on-orbit footprint camera calibration.

The results also show that there are obvious systematic errors among the data obtained from diverse orbits, and the RMSEs of the checkpoints are up to 3 pixels. Detailed analysis of the error sources is needed in further studies to eliminate the system errors automatically. In addition, the experiment images in this paper were all acquired by the footprint camera in single exposure mode, and there is no overlapping area between the adjacent images. In the mode of the three exposures, the overlap of first and third images in one period reaches up to 90%; thus, with the of auxiliary of high-precision DEM data, it is possible to explore the verification method based on two-image space intersection to verify the object positioning accuracy.

## 4. Conclusions

On-orbit geometric calibration is particularly important to ensure the follow-up application of footprint images of GF-7. This paper introduced the on-orbit geometric calibration model of the area-array footprint camera in detail, constructed a two-dimensional direction angle model, and used a polynomial model to fit the directional angles. Moreover, the validity and robustness of the calibration model constructed in this study were verified by the GF-7 satellite data. In order to solve the problem of the lack of control points for on-orbit calibration, a coarse-to-fine “LPM-SIFT + Phase correlation” matching strategy was proposed, which makes full use of the advantages of feature-based and region-based matching methods. Through the analysis and verification of the experimental data, the following conclusions can be summarized: (1) The matching results of the proposed method are reliable. High-quality and dense control points in even distribution can be obtained and the inner residuals of the matches is about 1/3 pixel in the image space. (2) The dense matches are conducive to the improvement of calibration accuracy. Compared with using the manual collected controls, the RMSE of the checkpoints has been improved from 1 pixel to less than 0.7 pixel in the single-image verification experiments. Therefore, the method proposed in this paper can be applied for the on-orbit calibration of the GF-7 footprint camera, and meets the high-frequency and efficient calibration requirements of the space-borne camera.

This study focuses on the single-image calibration method for the footprint camera of the GF-7 satellite. How to make full use of the laser altimeters and linear array cameras on the GF-7 satellite for joint calibration of multiple payloads will be further studied to fully understand the advantages of the new satellite and improve the overall accuracy of the remote sensing data.

## Figures and Tables

**Table 1:** Single-image calibration and verification results of the same orbit data.

Orbit-Image for Calibration	Controls for Calibration	RMSE of Controls (Pixel)	Image for Verification	Checkpoints	RMSE of Checkpoints (Pixel)
245–669 (California)	25	0.56	661	19	0.95
			664	22	0.87
			713	14	0.94
			**Mean value**		**0.92**
154–717 (Beijing)	25	0.55	719	10	0.98
			721	10	0.98
			**Mean value**		**0.98**

**Table 2:** Single-image verification results of different orbit data.

Experiments	Orbit -Image for Calibration	Orbit -Image for Verification	Checkpoints	RMSE of Checkpoints (Pixel)
Beijing → California	154–717	245–669	25	3.25
California → Beijing	245–669	154–717	25	3.17

**Table 3:** Matching and single-image calibration results.

Experiment Data	LPM-SIFT			PPFPR		
	Initial Matches	Mismatch Removal	RMSE (Pixel)	Initial Matches	Mismatch Removal	RMSE (Pixel)
**154–723**	223	204	**1.08**	584	580	**0.36**
**154–717**	142	93	**2.23**	586	581	**0.39**
		25 (Manually Collected)				**0.55**

**Table 4:** Mutual verification results based on dense matches.

Calibration Data		Verification Data		Results
Image for Calibration	Number of Controls	Image for Verification	Number of Checkpoints	RMSE of Checkpoints (Pixel)
**154–723**	580	**154–717**	581	**0.67**
**154–717**	581	**154–723**	580	**0.68**

## Data Availability

Data sharing not applicable.
